# Docosahexaenoic Acid Supplementation in Lactating Women Increases Breast Milk and Erythrocyte Membrane Docosahexaenoic Acid Concentrations and Alters Infant n–6:n–3 Fatty Acid Ratio

**DOI:** 10.1016/j.cdnut.2023.102010

**Published:** 2023-09-29

**Authors:** Shweta Khandelwal, Dimple Kondal, Ruby Gupta, Monica Chaudhry, Soumam Dutta, Lakshmy Ramakrishnan, Kamal Patil, M.K. Swamy, Dorairaj Prabhakaran, Nikhil Tandon, Usha Ramakrishnan, Aryeh D. Stein

**Affiliations:** 1 Department of Public Health Nutrition, Public Health Foundation of India, Gurugram, India; 2 Department of Biostatistics, Centre for Chronic Disease Control, Gurugram, India; 3 Department of Home Science, University of Calcutta, Kolkata, India; 4 Department of Nutrition, ICMR-National Institute of Nutrition, Hyderabad, India; 5 Department of Biochemistry, All India Institute of Medical Sciences (AIIMS), New Delhi, India; 6 Department of Obstetrics and Gynaecology, KLE’s J.N. Medical College Belagavi, Karnataka, India; 7 Hubert Department of Global Health, Rollins School of Public Health, Emory University, Atlanta, GA, United States

**Keywords:** long-chain polyunsaturated fatty acid (LC-PUFA), linoleic acid (LA), α linolenic acid (ALA), pregnancy, docosahexaenoic acid, fatty acid transfer, placenta, breast milk, n–3 LC-PUFA supplementation

## Abstract

**Background:**

Low concentrations of docosahexaenoic acid (DHA) or high n–6 (ω-6):n–3 ratio in pregnant women is associated with poor fetal growth velocity and suboptimal neurodevelopment. However, there is a lack of data on levels of important n–6 and n–3 fatty acids (FAs) at different time points during pregnancy and lactation from India. Data on how much DHA is transferred during actual supplementation are also scarce.

**Objectives:**

We report the concentrations of n–6 and n–3 FAs in maternal and infant blood and in breast milk following maternal supplementation with DHA or placebo.

**Methods:**

A total of 957 pregnant women (≤20 wk) from Belagavi, Karnataka, were randomly assigned to receive either 400 mg/d of algal DHA or placebo through 6 mo postpartum. Blood samples were collected from the mother at recruitment/baseline, delivery, and 6 mo postpartum and from the infant at birth (cord) and 12 mo (venous). Breast milk samples were collected from a subsample at delivery, 1 mo and 6 mo postpartum. The FA profile was analyzed using gas chromatography.

**Results:**

The concentration of DHA appeared to be higher in erythrocyte and breast milk samples of the DHA-supplemented group at all subsequent time points. The n–6:n–3 ratio was lower among women in the DHA group at delivery [DHA: 4.08 (1.79); placebo: 5.84 (3.57); *P* < 0.001] and at 6 mo postpartum [DHA: 5.34 (2.64); placebo: 7.69 (2.9); *P* < 0.001]. Infants of DHA-supplemented mothers also had a lower n–6:n–3 ratio at delivery and 12 mo. The n–6:n–3 ratio of breast milk increased from delivery through 1 to 6 mo but remained lower in the DHA-supplemented group than in the placebo.

**Conclusions:**

Maternal DHA supplementation with 400 mg/d from early pregnancy through 6 mo postpartum significantly increased circulating DHA in breast milk and infant erythrocyte, whereas decreased erythrocyte and breast milk n–6:n–3 ratio. However, maternal supplementation did not get the ratio to the recommended levels.

## Introduction

Maternal long-chain PUFAs (LC-PUFAs), including DHA (22:6n–3) and arachidonic acid (AA; 20:4n–6), are important for brain function and the development of the offspring, given their role in membrane structure, membrane fluidity, and neurotransmission [1,2]. DHA and AA are essential constituents of cell membrane phospholipids in the brain, and AA itself accounts for almost 20% of the total fatty acids (FAs) in neurons [3,4]. In extension to their role in brain function, maternal intake of LC-PUFAs, and their concentrations in the plasma of pregnant women have been linked to decreased postpartum depression rates, higher memory performance [5], and reduced learning disabilities in their newborns [[6], [7], [8]]*.* Modern diets, which are often deficient in PUFAs, increase individual susceptibility to diseases [[9], [10], [11]]. Although an intake of ≥250–500 mg/d of n–3 LC-PUFA is required to maintain physiologic needs for a healthy adult [3], the median dietary intakes of AA and DHA across 76 developing countries have been reported to be 64.0 and 48.9 mg/d, respectively [12]. Despite the available evidence, only a handful of countries have adopted guidelines to ensure optimum n–3 LC-PUFA intake in pregnant women [3].

A high n–6:n–3 LC-PUFA ratio has been associated with an increased risk of premenopausal breast cancer [13], increased adiposity and waist circumference, impaired metabolic profiles [14], dyslipidemia [15], and increased risk of mood disorders in young people [16]. Lower n–6:n–3 LC-PUFA ratio in pregnancy is positively associated with fetal growth velocity and duration of pregnancy [17] and lowers offspring systolic blood pressure [18]. Existing evidence suggests that a target n–6:n–3 FA ratio of 1:1–2:1 is associated with better health outcomes [19]. However, in India, the total dietary n–3 FA intake is low and the n–6:n–3 FA ratio has been reported to be 9 [20].

LC-PUFA concentrations increase markedly in the brain from the third trimester of pregnancy till 18 mo of life, and during this period, the offspring depends on the maternal LC-PUFA supply [21,22]. Maternal LC-PUFA concentrations, especially DHA and AA concentrations, decrease during pregnancy and their restoration to normal concentrations during the postpartum period is slow [23,24]. However, data on longitudinal variation in maternal and newborn LC-PUFA concentrations are scarce. Although a number of studies have reported LC-PUFA profiles during pregnancy [25] or in breast milk [[26], [27], [28]], limited literature is available for longitudinal variation in FA profiles in a mother–infant cohort [29]. To the best of our knowledge, this is the first study to assess longitudinal changes in DHA and the variation n–6:n–3 FA composition during pregnancy through 1 y postpartum across 4 different categories of samples (maternal blood, cord blood, breast milk, and infant blood). Moreover, we assess these changes within the context of a DHA supplementation trial.

## Methods

### Study population and design

DHA and Neurodevelopment in India (DHANI) was a randomized, double-blinded placebo-controlled trial designed to test the effect on offspring neurodevelopment of providing women with 400 mg/d algal DHA compared with placebo from ≤20 wk of singleton gestation through 6 mo postpartum. The trial protocol has been published elsewhere [30]. The protocol was reviewed and approved by the Institutional Review Board (IRBs) of all participating institutions: Center for Chronic Disease Control (CCDC-IEC_04_2015), Public Health Foundation of India (TRC-IEC-261/15), Jawaharlal Nehru Medical College (MDC/IECHSR/2016-17/A-85), and All India Institute of Medical Sciences (IEC-28/17.11.2015).

Healthy pregnant women (18–35 y; ≤20 wk of single gestation) attending the Department of Obstetrics and Gynecology at the Prabhakar Kore Hospital, Jawaharlal Nehru Medical College, Belagavi, Karnataka, India for their prenatal checkups were invited to participate. Consenting women were randomly assigned to receive either 400-mg DHA or a placebo till 6 mo postpartum. Assessments at enrolment included sociodemographic characteristics, dietary intake, obstetric history, anthropometric measurements, and blood investigations, including a nonfasting blood draw, and vital signs.

The intervention was either 400 mg/d algal DHA or a matching placebo (soy/corn oil) delivered in the form of similar-looking soft-gel capsules (DSM Nutritional Supplements). Enrolled women received their assigned supplement from the date of randomization until 6 mo postpartum.

### Collection of biological samples

FA composition of the phospholipids fraction from the red blood cell (RBC) membrane is an appropriate biomarker of FA status and reflects dietary intake in the previous few mo [31]. Maternal nonfasting blood samples (5 mL) were obtained by venipuncture at 3 time points—recruitment, delivery, and 6 mo postpartum. Neonatal blood samples were obtained from the umbilical cord vein immediately after delivery using the syringe method. A 2-mL venous blood sample was obtained from infants aged at 12 mo. All samples were collected into tubes containing disodium EDTA. Plasma was separated by cold centrifugation at 800 × *g* for 10 min at 4°C. RBCs were washed thrice using the same volume of saline as a packed cell in the tube. Washed RBCs were stored at −80°C for later analysis.

Breast milk samples were collected at child ages 1 d, 1 mo, and 6 mo, preferably in the morning hours. Infants were allowed to suckle for some time, after which the mother herself would express the milk into a sterile polypropylene container. After collecting sufficient volume (10 mL), feeding of the infant was resumed. The expressed milk was immediately stored at 4°C, and subsequently was aliquoted into tubes containing butylated hydroxytoluene and stored at −80°C for analysis.

### Analysis of biological samples

RBCs and breast milk samples were analyzed by gas chromatography using standard methods [32]. In short, lipids were extracted from the erythrocyte (RBCs) membrane using the Rose and Oklander method [33]; the phospholipid fraction was separated by thin-layer chromatography. FAs were esterified and analyzed on a 60 m column (DB-23, Agilent), using gas chromatography with a flame ionization detector (Agilent). Standards from Supelco (Sigma-Aldrich) and Nu-Chek (Nu-Chek Prep, Inc.) were used to identify FAs in the samples. The intra-assay and interassay coefficient of variation for all the FAs were <10%.

### Statistical analysis

We calculated total n–3 FAs by summing the values for α-linoleic acid (ALA, C18:3), EPA (C20:5), docosapentaenoic acid (C22:5), and DHA (C22:6). Total n–6 FAs were calculated as the sum of linoleic acid (LA, C18:2), γ-linoleic acid (GLA, 18:3), di-homo γ-linoleic acid (C20:3), and AA (C20:4). The n–6:n–3 ratio was calculated. EPA and docosapentaenoic acid (n–3) and GLA and di-homo γ-linoleic acid (n–6) were included for the calculation of n–6:n–3 ratio because they are intermediates during the conversion of ALA to DHA and LA to AA, respectively, and the essential FAs may be in the transition phase, depending on the metabolic state of the body.

The baseline characteristics of women at enrolment and infants at birth were presented as mean (SD) or median (25th percentile, 75th percentile) for continuous variables and number (%) for categorical variables. The FA concentrations (expressed in mol% of FA) in maternal erythrocyte at baseline, delivery, and 6 mo, in infant cord blood and venous sample at age 6 mo, and in breast milk at delivery,1 mo, and 6 mo were presented as mean (SD) and median [interquartile interval (p25, p75)].

We used the generalized estimating equation to assess within-group difference for the FA concentrations (calculated in mol% of FA) in maternal erythrocyte at baseline, delivery, and 6 mo and in infant cord blood and venous sample at age 6 mo. The *P* value of <0.05 was considered statistically significant. All the analysis was done using Stata 16.0.

## Results

A total of 957 pregnant women were recruited in the DHANI trial and data on 912 infants born were collected. A subsample of the biological samples (blood and breast milk) from these mother–child dyads were analyzed at several time points––baseline, delivery and 1 mo, 6 mo, and 12 mo postpartum. (Supplemental Figure 1 provides details of analyzed samples at each time point.)

At baseline, FA data were analyzed for *n* = 547 mothers (DHA = 291 and placebo; *n* = 256). FA data in breast milk were analyzed for a subsample (*n* = 120) of mothers at child ages 1 d, 1 mo, and 6 mo.

**FIGURE 1:**
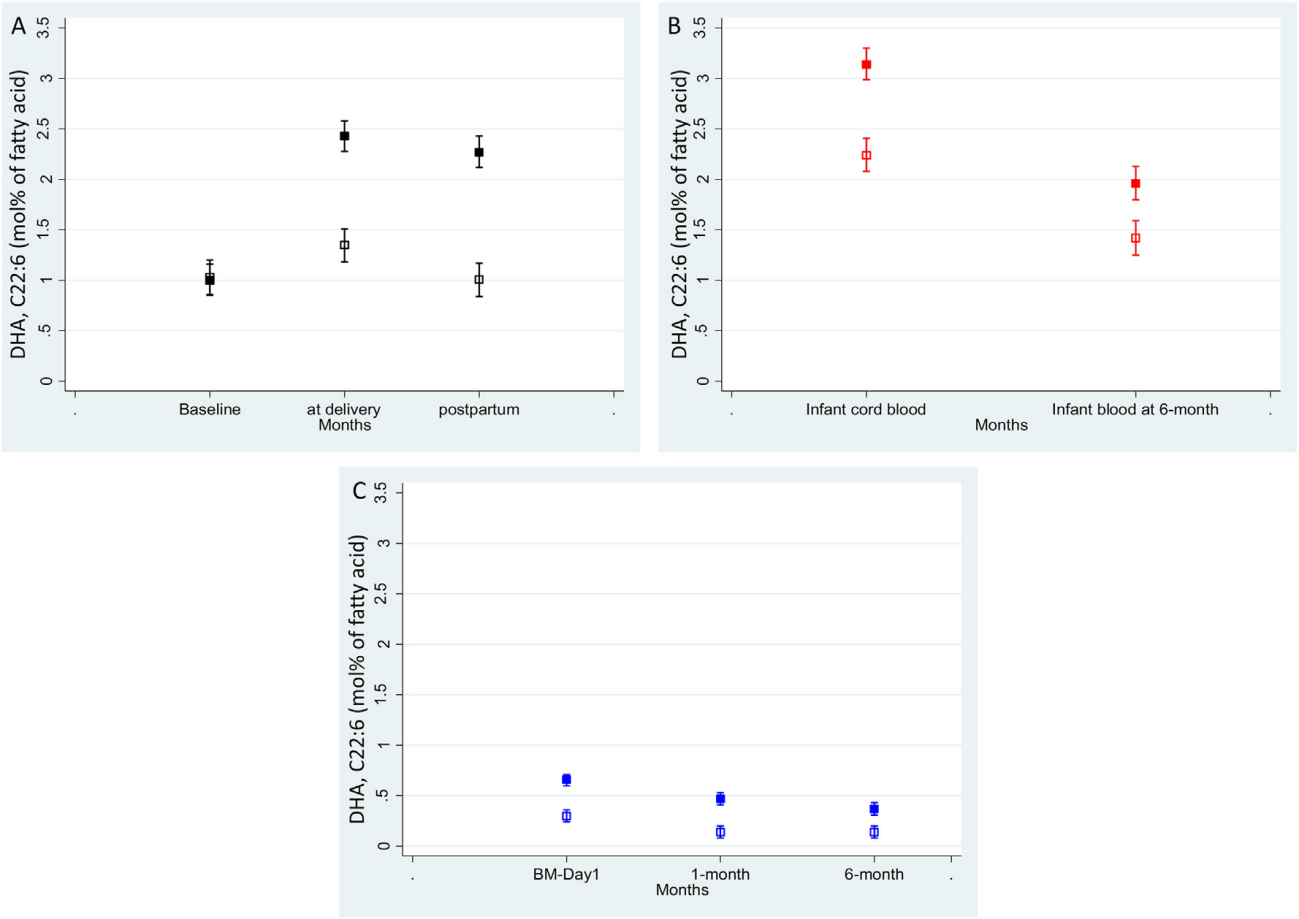
Mean DHA values (mol% of fatty acid) with 95% confidence interval in blood and breast milk samples, DHANI. (A) Maternal blood, (B) infant cord and venous blood, and (C) breast milk. Solid shapes for the DHA group and hollow shapes for the placebo group. DHANI, DHA and Neurodevelopment in India.

**FIGURE 2:**
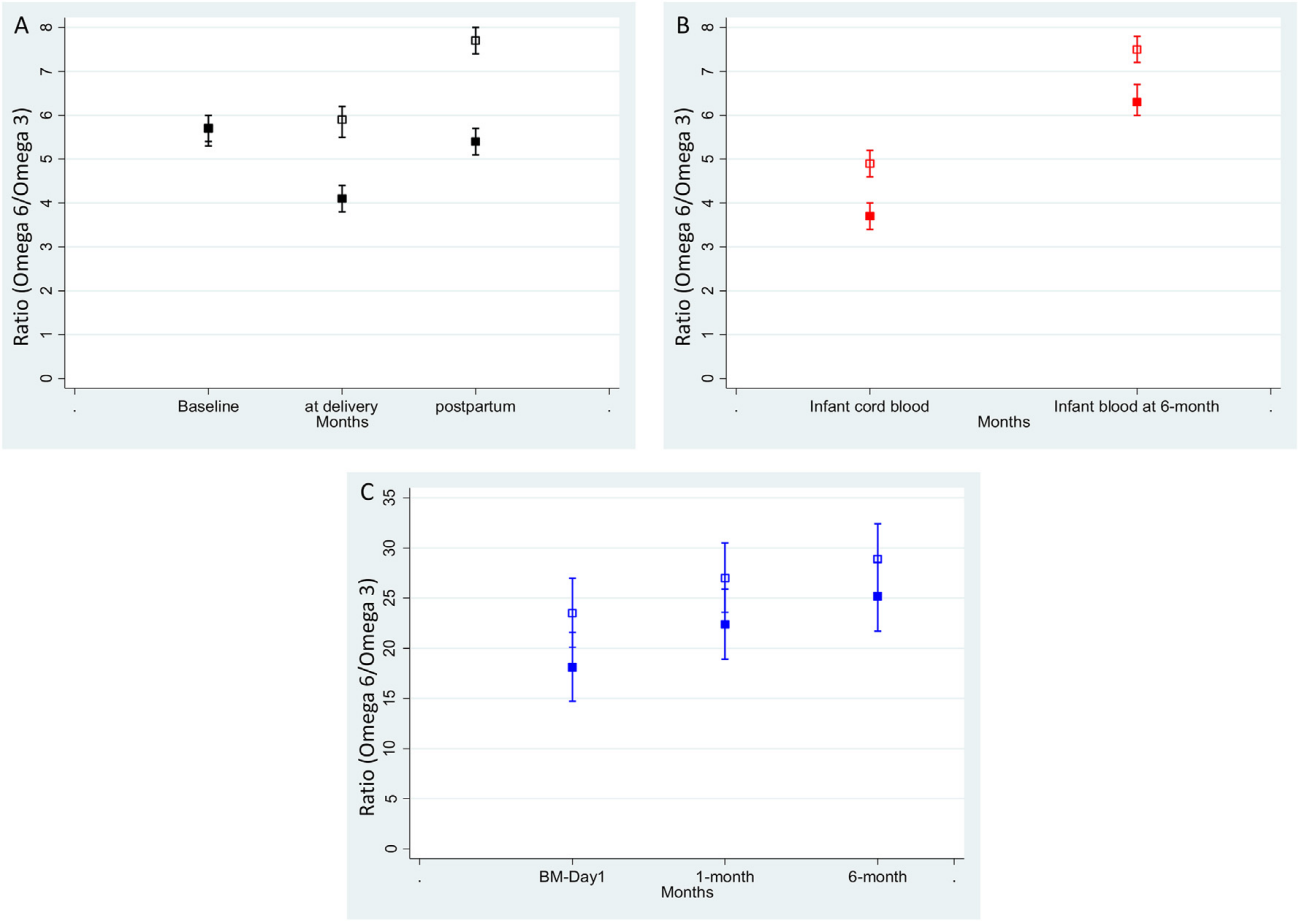
Mean ratio (omega-6/omega-3) with 95% confidence interval in blood and breast milk samples, DHANI. (A) Maternal blood, (B) infant cord and venous blood, and (C) breast milk. Solid shapes for the DHA group and hollow shapes for the placebo group. DHANI, DHA and Neurodevelopment in India.

Figure 1 presents the means and confidence intervals of DHA values (mol% of FA) values analyzed at different time points in the DHA and placebo groups. Figure 2 presents the means and confidence intervals of ratio (omega-6:omega-3) (mol% of FA) values analyzed at different time points in the DHA and placebo groups. Supplemental Figures 2–4 map the mean AA values, ω-6 values, and ω-3 values with 95% confidence interval in the DHA and placebo groups.

Table 1 presents the baseline characteristics of women at enrolment and infants at birth among mother–child dyads for which a blood sample, cord blood, or breast milk was available at any follow-up time points (*n* = 612). The mean age of the mothers was 23.5 (3.6) y. The gestational age at enrolment was 15.0 (12 and 18) wk. Overall, 85.8% of the women reported compliance of >90% based on pill counts.

**TABLE 1:** Baseline characteristics of women at enrolment and infants at birth among mother–child dyads, DHANI

	Overall (*n* = 612)	DHA (*n* = 320)	Placebo (*n* = 292)
Mother at enrolment			
Age (y)	23.6 (3.6)	23.6 (3.4)	23.6 (3.8)
Gestational age (wk)	15.0 (12.0, 18.0)	15.0 (12.0, 18.0)	15.0 (12.0, 18.0)
Household income, *n* (%)			
<10,000 INR	369 (60.3%)	189 (59.1%)	180 (61.6%)
10,001–20,000 INR	163 (26.6%)	81 (25.3%)	82 (28.1%)
>20,000 INR	67 (10.9%)	43 (13.4%)	24 (8.2%)
Do not know	13 (2.1%)	7 (2.2%)	6 (2.1%)
BMI (kg/m^2^), mean (SD)	20.6 (3.4)	20.7 (3.5)	20.5 (3.3)
SBP (mm Hg), mean (SD)	108.7 (10.4)	108.8 (10.5)	108.5 (10.3)
DBP (mm Hg), mean (SD)	68.3 (8.0)	68.7 (8.1)	67.9 (8.0)
Gestational age at the time of delivery (wk), median (p25, p 75)	38.9 (1.7)	38.8 (1.7)	38.9 (1.7)
Self-reported compliance	97.0 (94.0, 99.0)	97.0 (94.0, 98.0)	97.0 (93.0, 99.0)
Self-reported compliance (>90%)	516 (84.3)	277 (86.6)	239 (81.8)
Infant at birth			
Weight (g), mean (SD)	2772 (428)	2759 (413)	2786 (443)
Length (cm), mean (SD)	47.5 (2.0)	47.3 (2.0)	47.6 (2.0)
Head circumference (cm), mean (SD)	33.7 (1.4)	33.6 (1.4)	33.8 (1.4)

Abbreviations: DBP, diastolic blood pressure; DHANI, DHA and Neurodevelopment in India; SBP, systolic blood pressure.

Data expressed as mean (SD) or median (p25, p75), or *n* (%) as appropriate.

Includes women (and their children) for whom ≥1 blood sample was obtained after the infant was born.

### Maternal FA at baseline, delivery, and 6 mo

The maternal erythrocyte samples were analyzed at baseline, delivery, and 6 mo for *n* = 547, *n* = 587, and *n* = 564 women, respectively.

At baseline, there was no difference in mean DHA concentration (mol% of FA) between the DHA and placebo groups [DHA: 1.01 (0.95); placebo: 1.04 (0.86); *P* = 0.75] (Table 2). Similarly, there was no difference between the DHA and placebo group in mean AA [DHA: 5.35 (4.05); placebo: 5.50 (4.15); *P* = 0.69] or the n–6:n–3 ratio [DHA: 5.74 (2.76); placebo: 5.66 (3.29); *P* = 0.77].

**TABLE 2:** Fatty acid concentrations (expressed as mol% of fatty acid) in maternal blood at baseline, delivery, and 6 mo, DHANI

	Baseline	Delivery	6 mo postpartum	*P* value (within-group difference)
	Mean (SD)	Mean (SD)	Mean (SD)	
DHA (C22:6)				
DHA	1.01 (0.95)	2.43 (2.11)	2.28 (1.71)	<0.001
Placebo	1.04 (0.86)	1.35 (1.04)	1.00 (0.69)	0.0016
*P* value	0.838	<0.001	<0.001	
Arachidonic acid (AA, C20:4)				
DHA	5.35 (4.05)	5.68 (3.64)	7.82 (3.90)	<0.001
Placebo	5.50 (4.15)	6.47 (4.12)	8.78 (4.12)	<0.001
*P* value	0.717	0.018	0.003	
Total omega-6				
DHA	12.5 (5.95)	13.4 (5.38)	17.1 (5.62)	<0.001
Placebo	12.7 (6.15)	14.3 (5.86)	18.2 (5.93)	<0.001
*P* value	0.703	0.073	0.016	
Total omega-3				
DHA	2.55 (1.42)	3.85 (2.16)	3.78 (1.9)	0.206
Placebo	2.69 (1.39)	2.83 (1.34)	2.61 (1.12)	<0.001
*P* value	0.259	<0.001	0.556	
Omega-6:omega-3 ratio				
DHA	5.74 (2.76)	4.08 (1.79)	5.34 (2.64)	<0.001
Placebo	5.66 (3.29)	5.84 (3.57)	7.69 (2.9)	<0.001
*P* value	0.308	<0.001	<0.001	

Abbreviation: DHANI, DHA and Neurodevelopment in India.

Total omega-3: sum of α-linolenic acid, EPA, docosapentaenoic acid, and DHA; total omega-6: sum of linoleic acid, γ-linolenic acid, di-homo γ-linolenic acid, and arachidonic acid; baseline, DHA (*n* = 291), placebo (*n* = 256); delivery, DHA (*n* = 308), placebo (*n* = 279); 6 mo postpartum, DHA (*n* = 296), placebo (*n* = 268); *P* values calculated using a generalized estimating equation, the model includes treatment group, time, and interaction between the treatment group and time variable.

At delivery, the mean DHA value was higher in the DHA group than in placebo group [DHA: 2.43 (2.11); placebo: 1.35 (1.04); *P* < 0.001]. The mean n–6:n–3 ratio was lower in the DHA group than in the placebo group [DHA: 4.08 (1.79); placebo: 5.84 (3.57); *P* < 0.001]. Similarly, at 6 mo postpartum, the mean DHA values are higher in the DHA group than in the placebo group [DHA: 2.28 (1.71); placebo: 1.00 (0.69); *P* < 0.001].

### FA concentration in infant cord blood and venous sample at 12 mo

In infants, cord blood and age 12 mo samples were available for *n* = 575 and *n* = 507, respectively. The mean DHA values were higher in infant cord blood in the DHA group than in the placebo group [DHA: 3.14 (1.81); placebo: 2.25 (1.14); *P* ≤ 0.001] (Table 3). The mean AA was lower in infant cord blood in the DHA group than in the placebo group [DHA: 9.89 (4.1); placebo: 11.0 (4.1); *P* = 0.001]. The mean n–6:n–3 ratio in infant cord blood was lower in the DHA group than in the placebo group [DHA: 3.69 (1.47); placebo: 4.95 (2.24); *P* < 0.001].

**TABLE 3:** Fatty acid (mol% of fatty acid) in infant cord blood and venous blood at age 12 mo, DHANI

	Cord blood	Venous blood at age 12 mo	*P* value (within-group difference)
	Mean (SD)	Mean (SD)	
DHA (C22:6)			
DHA	3.14 (1.81)	1.99 (1.42)	<0.001
Placebo	2.25 (1.14)	1.43 (1.11)	<0.001
*P* value	<0.001	<0.001	
Arachidonic acid (AA, C20:4)			
DHA	9.89 (4.1)	8.81 (4.23)	0.0021
Placebo	11.0 (4.1)	9.03 (4.46)	<0.0001
*P* value	0.001	0.570	
Omega-6			
DHA	14.5 (4.32)	18.1 (5.98)	<0.001
Placebo	15.6 (4.27)	18.3 (6.15)	<0.001
*P* value	0.009	0.679	
Omega-3			
DHA	4.39 (1.83)	3.25 (1.64)	<0.001
Placebo	3.49 (1.24)	2.80 (1.44)	<0.001
*P* value	<0.001	0.001	
Omega-6:omega-3 ratio			
DHA	3.69 (1.47)	6.34 (2.17)	<0.001
Placebo	4.95 (2.24)	7.51 (2.82)	<0.001
*P* value	<0.001	<0.001	

Abbreviation: DHANI, DHA and Neurodevelopment in India.

Total omega-3 calculated as sum of α-linolenic acid, EPA, docosapentaenoic acid, and DHA; total omega-6 calculated as sum of linoleic acid, γ-linolenic acid, di-homo γ-linolenic acid, arachidonic acid; cord blood: DHA (*n* = 304), placebo (*n* = 271); venous blood at age 12 mo: DHA (*n* = 266), placebo (*n* = 241); *P* values calculated using a generalized estimating equation, the model includes treatment group, time and interaction between the treatment group and time variable.

At 12 mo, infant venous sample DHA was higher in the DHA-supplemented group than in the placebo group [DHA: 1.99 (1.42); placebo: 1.43 (1.11); *P* < 0.001]. The mean n–6:n–3 ratio was lower in the DHA group than in the placebo group [DHA: 6.34 (2.17); placebo: 7.51 (2.82); *P* < 0.001].

### FA in breast milk at day 1, 1 mo, and 6 mo

The FA in breast milk was analyzed for 120 mothers each at postdelivery day 1, 1 mo, and 6 mo. The baseline characteristics of these women (*n* = 120) were provided in Supplemental Table 1. The mean DHA values in breast milk at d 1 in the DHA group were higher than in the placebo group [DHA: 0.66 (0.31); placebo: 0.29 (0.17); *P* < 0.001] (Table 4). Similarly, the mean DHA values are higher in the DHA group at 1 mo [DHA: 0.47 (0.29); placebo: 0.14 (0.93); *P* < 0.001] and 6 mo [DHA: 0.37 (0.29); placebo: 0.14 (0.16); *P* < 0.001] than in the placebo group. The FA AA in breast milk was lower in the DHA group than in the control group over all time points (i.e., delivery, 1 mo, and 6 mo) than in the placebo group (*P* < 0.0001) (Table 4). Similarly, the n–6:n–3 ratio in breast milk was also lower in the DHA group than in the control group over all time points as compared with the placebo group (*P* < 0.0001) (Table 4).

**TABLE 4:** Fatty acid (expressed as mol% of fatty acid) in breast milk at postdelivery (day 1), 1 mo, and 6 mo, DHANI

	Delivery (day 1)	1 mo	6 mo	*P* value (within-group difference)
	Mean (SD)	Mean (SD)	Mean (SD)	
DHA (C22:6)				
DHA	0.66 (0.31)	0.47 (0.29)	0.37 (0.29)	<0.001
Placebo	0.29 (0.17)	0.14 (0.93)	0.14 (0.16)	<0.001
*P* value	<0.001	<0.001	<0.001	
Arachidonic acid (AA, C20:4)				
DHA	0.83 (0.21)	0.51 (0.12)	0.49 (0.14)	<0.001
Placebo	0.91 (0.20)	0.54 (0.14)	0.53 (0.12)	<0.001
*P* value	0.0073	0.2998	0.1719	
Omega- 6				
DHA	20.7 (2.9)	21.4 (5.16)	24.2 (4.99)	<0.001
Placebo	20.8 (3.9)	19.8 (6.14)	23.2 (5.84)	<0.001
*P* value	0.8451	0.0863	0.2736	
Omega-3				
DHA	1.3 (0.44)	1.26 (0.65)	1.33 (0.83)	0.6852
Placebo	0.99 (.35)	0.95 (0.55)	1.05 (0.65)	0.4698
*P* value	0.0048	0.0043	0.0103	
Omega-6:omega-3 ratio				
DHA	18.1 (7.92)	22.4 (14.7)	25.2 (16.3)	0.0005
Placebo	23.5 (10.1)	27 (16.1)	28.9 (15.9)	0.0106
*P* value	0.0317	0.0634	0.1368	

Abbreviation: DHANI, DHA and Neurodevelopment in India.

Total omega-3 constitutes sum of α-linolenic acid, EPA, docosapentaenoic acid, and DHA; total omega-6 constitutes sum of linoleic acid, γ-linolenic acid, di-homo γ-linolenic acid, arachidonic acid; DHA (*n* = 60), placebo (*n* = 60) at each time point. *P* values calculated using a generalized estimating equation, the model includes treatment group, time and interaction between the treatment group and time variable.

The complete FA profile of maternal blood, infant blood, and breast milk samples at different time points is presented in Supplemental Table 2.

## Discussion

In this study, we provide novel longitudinal data on maternal and infant LC-PUFA profile in the context of a supplementation trial with 400 mg/d DHA. We observed a significant increase in DHA concentrations and a decrease in the n–6:n–3 FA ratio in the erythrocyte membrane of mother–infant dyads and in breast milk of the supplemented group as compared with the placebo group. In addition, we observed a decrease in erythrocyte membrane and breast milk AA concentrations in the DHA-supplemented group.

Two previous studies have assessed the longitudinal variation of FA profile in a post–LC-PUFA supplementation cohort; however, in those studies, supplementation was only provided during the lactation period [26,34]. To the best our knowledge, this is the first study to report changes in n–6:n–3 ratio starting from mid-pregnancy through to 1 y postpartum across 4 biological samples (maternal blood, cord blood, breast milk, and infant blood).

Studies from elsewhere note that although the concentrations of n–6 PUFA increase from the first to the third trimester of pregnancy, AA decreases [35]. At the same time, n–3 PUFA and EPA concentrations decreased during pregnancy, whereas DHA concentrations mostly remained stable [35]. In high-income countries, LC-PUFA concentrations decrease from the second to the third trimester [36,37] and the DHA deficiency index increases [38]. DHA concentrations in mothers decrease during pregnancy and postpartum period, reach a minimum at 6 mo after birth, and return to prepregnancy levels within 1 y [33]. The decrease in maternal DHA concentrations may be attributed to accretion of DHA by the offspring via placental transfer and lactation, in the absence of adequate maternal intakes [29].

The n–6:n–3 ratio serves as an important indicator of FA profile. Alteration in this ratio is associated with several chronic inflammatory conditions as well as noncommunicable diseases. Maternal n–6:n–3 ratio mostly depends on the dietary intake [39]. Nondietary factors, such as FA supply from the maternal body to the fetus through the placenta [40] are also important. Modern dietary patterns result in dietary n–6:n–3 ratios as high as 20–30:1 [39] Perinatal exposure to high concentrations of n–6 FAs may influence the health and nutritional status of the infants. For instance, adverse maternal FA profile, higher concentrations of n–6 and lower concentrations of n–3 FAs are associated with reduced fetal growth [41]. Increased concentrations of n–6 FAs in the second trimester of pregnancy are associated with higher odds of atopic dermatitis in children, although there was no significant relation with prenatal n–6:n–3 ratio [42]. Similarly dietary n–6:n–3 ratio during pregnancy is negatively associated with mental and psychomotor development in infants at 6 mo [43]. Higher n–6:n–3 fatty ratio (AA/EPA + DHA) in breast milk has been associated with increased infant adipogenesis [44]. Although a ratio of 2:1 is considered ideal [45], the typical ratio in India is ∼9:1 or more. In our study, it was 5 at baseline, and supplementation with 400 mg/dL DHA was able to shift the ratio only to a limited extent. Other strategies, such as a higher dose of supplementation or reducing the intake of n–6 FAs etc., may be needed to get closer to the optimal ratio.

Furthermore, during the course of lactation, the ratio of n–6:n–3 FAs in maternal erythrocytes increases but the concentration of DHA in breast milk remains more or less constant, suggesting that DHA continues to be transferred to the infants through breast milk [39]. Thus, adequate breastfeeding may compensate for prenatal imbalances in maternal dietary n–6:n–3 ratio [45]. DHA concentrations in human milk vary across countries and regions, with low concentrations in Canada and the United States and higher concentrations in Japan [46]. DHA concentrations in breast milk decrease from 0.5% in colostrum to 0.25% in mature milk [[47], [48], [49]]*.* Breastfeeding women need to consume ≥200-mg DHA per day to produce milk with a DHA content of a minimum of 0.3%. This DHA concentration is essential for a breastfed infant to obtain an adequate DHA supply (100 mg DHA/d) to meet its metabolic needs [29].

However, this study has a few limitations that should be acknowledged. Genetic factors, such as single-nucleotide polymorphisms in the FADS gene could not be studied that may determine who will be benefited from supplementation [50]. Data for the pro- and anti-inflammatory cytokine profile of the mother–infant dyads, which is associated with n–6:n–3 ratio, are not available. Future large-scale studies should consider all these factors. On the other hand, some of the key strengths of this study include a strong study design, large sample size, high retention rate, and excellent compliance.

In conclusion, this study indicates that maternal DHA supplementation from mid-pregnancy through 6 mo postpartum increased maternal and infant erythrocyte and breast milk DHA concentrations, along with a decrease in AA concentrations that persisted through infant age 12 mo. However, the n–6:n–3 ratio in erythrocyte or milk did not approach 2, a value thought to be optimal for human health, and even higher doses may be required to achieve this goal. This evidence may guide future public health nutrition studies to test higher dosages of DHA consumption and/or supplementation and assess their role in maternal and child health.

## Author contributions

The authors’ responsibilities were as follows – SK, ADS, UR, NT, DP: concept and design; SK, DK, RG, ADS: acquisition, analysis, or interpretation of data; SK, DK, MC, SD: drafting of the manuscript; ADS, DP, NT, UR: critical revision of the manuscript for important intellectual content; DK: statistical analysis; MC, MKS, NT, DP: administrative, technical, or material support; KP, ADS, NT, DP: supervision; SK: had full access to all the data in the study and took responsibility for the integrity of the data and the accuracy of the data analysis; and all authors: read and approved the final manuscript.

## Conflict of interest

ADS is a member of the Journal’s Editorial Board and a 2022 fellow of ASN. All other authors report no conflicts of interest.

## Funding

This work was supported by Wellcome Trust-DBT India Alliance (Grant Number: IA/CPHE/14/1/501498). The supplements were donated by DSM Mumbai, India. The funding sources had no role in the design and conduct of the study; collection, management, analysis, and interpretation of the data; preparation, review, or approval of the manuscript; and decision to submit the manuscript for publication. Neither the product provider nor the sponsors had an opportunity to review a presubmission copy of the article. SK and DP obtained funding.

## Data availability

Data described in the manuscript, code book, and analytic code will be made available upon reasonable request via e-mail to the corresponding author.

## Declaration of interests

The authors declare the following financial interests/personal relationships which may be considered as potential competing interests: Shweta Khandelwal reports financial support was provided by Wellcome Trust DBT India Alliance.

## Appendix A Supplementary data

Multimedia component1 Multimedia component1
