# Supplementary material for: Docosahexaenoic Acid Supplementation in Lactating Women Increases Breast Milk and Erythrocyte Membrane Docosahexaenoic Acid Concentrations and Alters Infant n–6:n–3 Fatty Acid Ratio
Source: Curr Dev Nutr. 2023 Sep 29;7(10):102010. doi: 10.1016/j.cdnut.2023.102010 (PMC10590723; doi:10.1016/j.cdnut.2023.102010)
Supplement: Multimedia component1 [file mmc1.docx]

**Docosahexaenoic acid supplementation in lactating women increases breast milk and erythrocyte membrane docosahexaenoic acid concentrations and**

**alters infant n-6: n-3 fatty acid ratio**

Dr Shweta Khandelwal

Table S1: Baseline characteristics of a subsample of women whose breast milk samples were analyzed from DHANI cohort

|  | **Overall**  **(n=120)** | **DHA**  **(n=60)** | **Placebo**  **(n=60)** |
| --- | --- | --- | --- |
| Mother at enrolment |  |  |  |
| Age (year), mean(SD) | 23.2 (3.6) | 23.4 (3.1) | 23.1 (4.0) |
| Gestational age (weeks), median (p25, p 75) | 14.0 (12.0, 18.0) | 14.0 (12.0, 18.5) | 14.0 (12.0, 18.0) |
| Household income, n(%) |  |  |  |
| *<10000 INR* | 79 (65.8%) | 36 (60%) | 43 (72%) |
| *10,001-20,000 INR* | 25 (20.8%) | 11 (18%) | 14 (23%) |
| *>20000 INR* | 14 (11.7%) | 11 (18%) | 3 (5%) |
| *Don’t Know* | 2 (1.7%) | 2 (3%) | 0 (0%) |
| Body Mass Index (kg/m^2^), mean(SD) | 20.3 (2.9) | 19.8 (2.6) | 20.8 (3.1) |
| Systolic Blood Pressure (mmHg), mean(SD) | 109.7 (10.6) | 109.7 (10.3) | 109.6 (10.9) |
| Diastolic Blood Pressure (mmHg), mean(SD) | 68.9 (8.7) | 69.3 (8.8) | 68.6 (8.7) |
| Gestational age at the time of delivery (weeks), median (p25, p 75)* | 38.9 (1.7) | 38.8 (1.8) | 38.9 (1.7) |
| Self-reported compliance, median (p25, p75) | 97.0 (94.0, 98.0) | 96.5 (94.0, 98.0) | 97.0 (94.5, 99.0) |
| Self-reported compliance (>90%) | 103 (85.8) | 53 (88.0) | 50 (83.0) |
| Infant at birth |  |  |  |
| Weight (gram), mean(SD) | 2728.0 (401.9) | 2702.0 (396.1) | 2753.0 (409.2) |
| Length (cm), mean(SD) | 47.3 (1.9) | 47.2 (2.0) | 47.5 (1.8) |
| Head circumference (cm), mean(SD) | 33.5 (1.5) | 33.4 (1.5) | 33.5 (1.5) |

Table S2: Complete fatty acid profile in maternal blood at baseline, delivery, and 6-month; infant cord blood and venous blood at age 12 months; breast milk (day-1), 1-month and 6-month. Data expressed as Mean (SD) and Median (P25, P75) (mol % of fatty acid)

|  | **Maternal blood-Baseline** | **Maternal blood-Delivery** | **Maternal blood-6 month** | **Infant-cord blood** | **Infant-Venous blood at 12m** | **Breastmilk-Delivery (day 1)** | | **Breastmilk-1month** | **Breastmilk-6month** |
| --- | --- | --- | --- | --- | --- | --- | --- | --- | --- |
| **Variable(s)** |  |  |  |  |  |  | |  |  |
| **Docosahexaenoic acid, n3** |  |  |  |  |  |  | |  |  |
| DHA *mean (SD)* | 1.01 (0.95) | 2.43 (2.11) | 2.28 (1.71) | 3.14 (1.81) | 1.99 (1.42) | 0.66 (0.31) | | 0.47 (0.30) | 0.37 (0.29) |
| *median (IQR)* | 0.63 (0.36, 1.38) | 1.72 (0.74, 3.58) | 1.92 (0.79, 3.60) | 3.10 (1.58, 4.63) | 1.73 (0.68, 3.08) | 0.66 (0.38, 0.90) | | 0.38 (0.22, 0.74) | 0.29 (0.14, 0.56) |
| Placebo  *mean (SD)* | 1.04 (0.86) | 1.35 (1.04) | 1.00 (0.69) | 2.25 (1.14) | 1.43 (1.11) | 0.30 (0.17) | | 0.14 (0.09) | 0.14 (0.16) |
| *median (IQR)* | 0.64 (0.41, 1.53) | 0.98 (0.51, 2.10) | 0.89 (0.47, 1.35) | 2.15 (1.36, 3.11) | 1.07 (0.51, 2.08) | 0.25 (0.19, 0.34) | | 0.12 (0.10, 0.16) | 0.10 (0.07, 0.15) |
| **Lauric acid** |  |  |  |  |  |  | |  |  |
| DHA *mean (SD)* | 1.51 (1.77) | 1.23 (1.34) | 0.93 (0.76) | 1.01 (0.89) | 0.58 (0.43) | 3.44 (2.37) | | 8.95 (3.23) | 7.55 (2.07) |
| *median (IQR)* | 0.91 (0.46, 1.93) | 0.85 (0.44, 1.63) | 0.77 (0.47, 1.12) | 0.73 (0.43, 1.22) | 0.47 (0.27, 0.75) | 2.69 (2.11, 3.78) | | 8.56 (6.43, 11.00) | 7.82 (5.81, 8.91) |
| Placebo  *mean (SD)* | 1.43 (1.45) | 1.28 (1.38) | 0.96 (1.12) | 0.97 (0.75) | 0.61 (0.46) | 3.11 (1.88) | | 9.48 (2.76) | 7.56 (2.01) |
| *median (IQR)* | 0.97 (0.48, 1.77) | 0.87 (0.46, 1.53) | 0.79 (0.50, 1.11) | 0.75 (0.43, 1.29) | 0.52 (0.30, 0.81) | 2.71 (1.96, 3.49) | | 9.44 (7.47, 11.30) | 7.34 (6.09, 8.72) |
| **Myristic acid** |  |  |  |  |  |  | |  |  |
| DHA *mean (SD)* | 1.45 (1.48) | 1.24 (1.15) | 1.17 (0.95) | 1.07 (0.85) | 0.76 (0.33) | 5.09 (1.61) | | 8.43 (2.80) | 7.66 (2.31) |
| *median (IQR)* | 0.80 (0.56, 1.92) | 0.77 (0.55, 1.69) | 0.83 (0.55, 1.58) | 0.72 (0.55, 1.22) | 0.70 (0.56, 0.90) | 4.76 (3.96, 5.62) | | 8.30 (6.32, 10.40) | 7.56 (5.74, 9.09) |
| Placebo  *mean (SD)* | 1.47 (1.54) | 1.30 (1.36) | 1.09 (1.13) | 1.09 (0.88) | 0.74 (0.32) | 5.04 (1.71) | | 9.50 (2.67) | 8.01 (2.33) |
| *median (IQR)* | 0.81 (0.57, 1.76) | 0.75 (0.53, 1.55) | 0.75 (0.56, 1.37) | 0.71 (0.53, 1.45) | 0.68 (0.56, 0.86) | 4.69 (4.08, 5.30) | | 9.18 (7.43, 11.20) | 7.78 (6.03, 9.65) |
| Myristoleic acid |  |  |  |  |  |  | |  |  |
| DHA *mean (SD)* | 0.34 (0.35) | 0.28 (0.25) | 0.34 (0.43) | 0.29 (0.32) | 0.33 (0.56) | 0.11 (0.12) | | 0.19 (0.08) | 0.14 (0.07) |
| *median (IQR)* | 0.27 (0.16, 0.40) | 0.23 (0.15, 0.33) | 0.24 (0.17, 0.35) | 0.21 (0.14, 0.32) | 0.18 (0.12, 0.30) | 0.09 (0.08, 0.12) | | 0.17 (0.13, 0.24) | 0.13 (0.09, 0.15) |
| Placebo  *mean (SD)* | 0.39 (0.53) | 0.34 (0.60) | 0.29 (0.31) | 0.28 (0.29) | 0.33 (0.56) | 0.10 (0.04) | | 0.25 (0.26) | 0.15 (0.07) |
| *median (IQR)* | 0.25 (0.16, 0.43) | 0.22 (0.16, 0.34) | 0.23 (0.15, 0.34) | 0.21 (0.15, 0.31) | 0.20 (0.12, 0.32) | 0.09 (0.07, 0.12) | | 0.20 (0.15, 0.26) | 0.14 (0.10, 0.18) |
| **Pentadecanoic acid** |  |  |  |  |  |  | |  |  |
| DHA *mean (SD)* | 0.48 (1.13) | 0.41 (0.54) | 0.33 (0.16) | 0.35 (0.28) | 0.27 (0.16) | 0.23 (0.07) | | 0.26 (0.11) | 0.21 (0.08) |
| *median (IQR)* | 0.33 (0.26, 0.44) | 0.30 (0.23, 0.42) | 0.29 (0.22, 0.40) | 0.28 (0.21, 0.36) | 0.24 (0.19, 0.31) | 0.20 (0.18, 0.25) | | 0.23 (0.18, 0.34) | 0.20 (0.16, 0.25) |
| Placebo  *mean (SD)* | 0.47 (0.72) | 0.38 (0.31) | 0.31 (0.12) | 0.32 (0.24) | 0.26 (0.12) | 0.23 (0.06) | | 0.29 (0.15) | 0.22 (0.08) |
| *median (IQR)* | 0.35 (0.26, 0.47) | 0.31 (0.22, 0.41) | 0.30 (0.23, 0.37) | 0.26 (0.20, 0.34) | 0.24 (0.17, 0.32) | 0.22 (0.19, 0.26) | | 0.24 (0.20, 0.33) | 0.21 (0.15, 0.27) |
| **Pentadecenoic acid** |  |  |  |  |  |  | |  |  |
| DHA *mean (SD)* | 2.42 (2.05) | 2.48 (2.55) | 2.25 (1.58) | 2.14 (1.94) | 1.51 (0.81) | 0.26 (0.20) | | 0.15 (0.07) | 0.19 (0.13) |
| *median (IQR)* | 1.75 (1.02, 2.91) | 1.59 (0.93, 3.11) | 2.04 (1.09, 2.75) | 1.61 (0.83, 2.80) | 1.36 (0.90, 2.01) | 0.21 (0.16, 0.29) | | 0.14 (0.11, 0.20) | 0.16 (0.11, 0.23) |
| Placebo  *mean (SD)* | 2.55 (2.31) | 2.36 (2.00) | 2.38 (1.75) | 1.99 (1.68) | 1.72 (0.97) | 0.26 (0.18) | | 0.15 (0.07) | 0.18 (0.12) |
| *median (IQR)* | 1.81 (0.99, 3.03) | 1.65 (0.88, 3.16) | 2.16 (1.21, 2.85) | 1.56 (0.82, 2.36) | 1.49 (1.09, 2.15) | 0.21 (0.13, 0.33) | | 0.14 (0.10, 0.19) | 0.15 (0.11, 0.20) |
| **Palmitic acid** |  |  |  |  |  |  | |  |  |
| DHA *mean (SD)* | 30.6 (4.89) | 30.4 (4.89) | 28.0 (4.52) | 29.7 (3.92) | 28.0 (3.98) | 25.7 (2.85) | | 22.8 (3.34) | 21.9 (3.28) |
| *median (IQR)* | 31.3 (26.5, 34.3) | 30.5 (27.0, 33.9) | 26.8 (24.0, 31.7) | 29.1 (26.8, 32.2) | 28.1 (24.5, 31.1) | 25.9 (24.6, 27.0) | | 23.1 (20.3, 25.3) | 22.3 (19.7, 24.0) |
| Placebo  *mean (SD)* | 30.2 (4.44) | 30.4 (5.12) | 27.5 (4.42) | 29.3 (3.69) | 28.0 (4.04) | 26.1 (2.13) | | 23.4 (3.63) | 22.6 (3.48) |
| *median (IQR)* | 30.3 (26.2, 33.9) | 30.1 (26.8, 33.9) | 26.3 (24.1, 31.2) | 29.0 (26.6, 31.5) | 28.5 (24.5, 31.1) | 26.0 (24.8, 27.5) | | 23.2 (20.8, 26.0) | 22.3 (19.5, 25.6) |
| Palmoleic acid |  |  |  |  |  |  | |  |  |
| DHA *mean (SD)* | 0.60 (0.38) | 0.57 (0.35) | 0.67 (1.56) | 0.67 (0.30) | 0.47 (0.53) | 1.86 (0.49) | | 2.31 (0.95) | 1.97 (0.86) |
| *median (IQR)* | 0.52 (0.34, 0.72) | 0.49 (0.35, 0.70) | 0.42 (0.28, 0.58) | 0.63 (0.47, 0.84) | 0.30 (0.20, 0.51) | 1.87 (1.50, 2.24) | | 2.09 (1.73, 2.70) | 1.88 (1.33, 2.48) |
| Placebo  *mean (SD)* | 0.61 (0.46) | 0.58 (0.39) | 0.59 (0.93) | 0.75 (0.66) | 0.66 (2.39) | 1.96 (0.69) | | 2.44 (1.06) | 2.13 (0.89) |
| *median (IQR)* | 0.49 (0.34, 0.72) | 0.49 (0.35, 0.67) | 0.40 (0.29, 0.56) | 0.65 (0.49, 0.86) | 0.34 (0.23, 0.53) | 1.88 (1.47, 2.32) | | 2.19 (1.71, 2.96) | 1.91 (1.45, 2.78) |
| **Stearic acid** |  |  |  |  |  |  | |  |  |
| DHA *mean (SD)* | 15.3 (2.09) | 14.3 (1.96) | 16.0 (2.02) | 16.2 (2.03) | 17.7 (2.31) | 4.99 (0.77) | | 4.78 (0.87) | 4.61 (0.76) |
| *median (IQR)* | 15.4 (14.2, 16.6) | 14.2 (13.2, 15.5) | 15.8 (14.7, 17.1) | 16.1 (15.0, 17.4) | 17.4 (16.2, 18.9) | 5.06 (4.65, 5.51) | | 4.64 (4.10, 5.46) | 4.46 (4.16, 4.94) |
| Placebo  *mean (SD)* | 15.2 (2.37) | 14.4 (2.27) | 16.1 (2.14) | 16.2 (1.82) | 17.7 (2.42) | 5.02 (0.79) | | 4.85 (1.09) | 4.60 (0.76) |
| *median (IQR)* | 15.1 (13.7, 16.6) | 14.4 (13.2, 15.6) | 15.6 (14.7, 17.8) | 16.1 (15.0, 17.3) | 17.4 (16.3, 19.0) | 4.97 (4.5, 5.51) | | 4.54 (4.0, 5.6) | 4.65 (4.02, 5.16) |
| **Elaidic acid** |  |  |  |  |  |  | |  |  |
| DHA *mean (SD)* | 0.34 (0.29) | 0.32 (0.29) | 0.33 (0.64) | 0.29 (0.25) | 0.22 (0.49) | 0.19 (0.09) | | 0.18 (0.11) | 0.16 (0.11) |
| *median (IQR)* | 0.27 (0.17, 0.44) | 0.24 (0.15, 0.39) | 0.24 (0.18, 0.35) | 0.23 (0.14, 0.35) | 0.17 (0.12, 0.24) | 0.18 (0.13, 0.24) | | 0.17 (0.11, 0.24) | 0.13 (0.09, 0.21) |
| Placebo  *mean (SD)* | 0.40 (0.62) | 0.35 (0.48) | 0.33 (0.65) | 0.30 (0.33) | 0.26 (0.67) | 0.19 (0.10) | | 0.20 (0.17) | 0.17 (0.11) |
| *median (IQR)* | 0.29 (0.18, 0.46) | 0.25 (0.18, 0.39) | 0.26 (0.17, 0.38) | 0.22 (0.14, 0.35) | 0.17 (0.13, 0.27) | 0.15 (0.13, 0.23) | | 0.16 (0.11, 0.26) | 0.17 (0.08, 0.21) |
| **Oleic acid** |  |  |  |  |  |  | |  |  |
| DHA *mean (SD)* | 11.00 (1.89) | 11.20 (2.06) | 10.30 (1.72) | 9.47 (1.70) | 10.10 (1.87) | 31.70 (3.17) | | 27.60 (3.89) | 28.50 (3.33) |
| *median (IQR)* | 11.10 (10.10, 12.00) | 11.60 (10.40, 12.40) | 10.40 (9.42, 11.30) | 9.41 (8.55, 10.50) | 10.30 (9.40, 11.30) | 32.60 (29.50, 34.20) | | 27.20 (25.00, 30.50) | 28.50 (26.10, 30.50) |
| Placebo  *mean (SD)* | 10.70 (1.95) | 11.10 (3.05) | 10.30 (1.70) | 9.40 (1.48) | 10.10 (1.87) | 31.70 (3.09) | | 27.00 (3.98) | 28.60 (3.44) |
| *median (IQR)* | 10.90 (9.85, 12.00) | 11.30 (10.10, 12.20) | 10.40 (9.46, 11.30) | 9.40 (8.63, 10.30) | 10.30 (9.38, 11.20) | 31.50 (29.90, 33.80) | | 26.80 (24.40, 29.30) | 27.90 (26.30, 30.70) |
| **Linoelaidic acid** |  |  |  |  |  |  | |  |  |
| DHA *mean (SD)* | 0.31 (0.20) | 0.32 (0.35) | 0.29 (0.16) | 0.30 (0.28) | 0.21 (0.10) | 0.05 (0.04) | | 0.03 (0.03) | 0.04 (0.04) |
| *median (IQR)* | 0.26 (0.18, 0.39) | 0.24 (0.17, 0.37) | 0.25 (0.18, 0.35) | 0.25 (0.17, 0.36) | 0.19 (0.14, 0.26) | 0.03 (0.02, 0.05) | | 0.02 (0.02, 0.04) | 0.03 (0.02, 0.05) |
| Placebo  *mean (SD)* | 0.31 (0.18) | 0.32 (0.28) | 0.29 (0.15) | 0.28 (0.18) | 0.22 (0.11) | 0.06 (0.09) | | 0.04 (0.04) | 0.04 (0.04) |
| *median (IQR)* | 0.26 (0.18, 0.44) | 0.25 (0.19, 0.37) | 0.26 (0.18, 0.35) | 0.24 (0.17, 0.33) | 0.20 (0.15, 0.28) | 0.03 (0.02, 0.06) | | 0.03 (0.02, 0.05) | 0.03 (0.01, 0.04) |
| **Linoleic acid, n6** |  |  |  |  |  |  | |  |  |
| DHA *mean (SD)* | 6.42 (2.38) | 7.07 (2.41) | 8.50 (2.28) | 3.78 (1.57) | 8.72 (2.39) | 18.90 (2.95) | | 20.00 (5.19) | 23.10 (4.99) |
| *median (IQR)* | 6.16 (4.44, 8.24) | 7.22 (5.22, 8.89) | 8.65 (6.94, 10.10) | 3.52 (3.03, 4.07) | 8.82 (7.17, 10.30) | 18.60 (16.90, 20.80) | | 19.90 (16.40, 23.30) | 23.60 (21.00, 26.20) |
| Placebo  *mean (SD)* | 6.43 (2.69) | 7.11 (2.71) | 8.69 (2.57) | 3.86 (1.67) | 8.70 (2.37) | 19.00 (3.86) | | 18.40 (6.09) | 22.10 (5.81) |
| *median (IQR)* | 6.45 (4.40, 8.32) | 7.19 (5.03, 8.87) | 8.94 (7.13, 10.30) | 3.53 (3.04, 4.02) | 8.96 (7.07, 10.40) | 18.80 (16.20, 21.50) | | 18.80 (13.50, 21.40) | 21.40 (18.30, 26.60) |
| **Gamma linoleic acid, n6** |  |  |  |  |  |  | |  |  |
| DHA *mean (SD)* | 0.38 (0.27) | 0.34 (0.26) | 0.34 (0.18) | 0.34 (0.27) | 0.31 (0.24) | 0.07 (0.05) | | 0.18 (0.06) | 0.15 (0.06) |
| *median (IQR)* | 0.32 (0.22, 0.45) | 0.28 (0.20, 0.41) | 0.28 (0.21, 0.44) | 0.27 (0.19, 0.40) | 0.25 (0.18, 0.36) | 0.05 (0.04, 0.09) | | 0.18 (0.14, 0.22) | 0.15 (0.10, 0.19) |
| Placebo  *mean (SD)* | 0.41 (0.27) | 0.37 (0.29) | 0.33 (0.20) | 0.34 (0.27) | 0.31 (0.22) | 0.08 (0.05) | | 0.20 (0.08) | 0.17 (0.06) |
| *median (IQR)* | 0.34 (0.23, 0.50) | 0.30 (0.21, 0.42) | 0.28 (0.20, 0.39) | 0.27 (0.18, 0.40) | 0.24 (0.17, 0.38) | 0.07 (0.05, 0.11) | | 0.19 (0.15, 0.23) | 0.16 (0.12, 0.21) |
| **Alpha linoleic acid, n3** |  |  |  |  |  |  | |  |  |
| DHA *mean (SD)* | 0.40 (0.24) | 0.37 (0.24) | 0.41 (0.26) | 0.34 (0.18) | 0.29 (0.19) | 0.45 (0.22) | | 0.65 (0.51) | 0.83 (0.56) |
| *median (IQR)* | 0.37 (0.22, 0.51) | 0.31 (0.23, 0.47) | 0.34 (0.23, 0.50) | 0.30 (0.20, 0.43) | 0.24 (0.16, 0.35) | 0.40 (0.28, 0.55) | | 0.45 (0.32, 0.74) | 0.46 (0.35, 1.32) |
| Placebo  *mean (SD)* | 0.43 (0.27) | 0.39 (0.25) | 0.38 (0.20) | 0.34 (0.20) | 0.29 (0.20) | 0.49 (0.23) | | 0.66 (0.51) | 0.77 (0.56) |
| *median (IQR)* | 0.37 (0.24, 0.57) | 0.31 (0.23, 0.48) | 0.33 (0.23, 0.48) | 0.29 (0.20, 0.41) | 0.23 (0.16, 0.36) | 0.45 (0.32, 0.61) | | 0.38 (0.30, 0.90) | 0.52 (0.37, 0.97) |
| **Arachidic acid** |  |  |  |  |  |  | |  |  |
| DHA *mean (SD)* | 0.68 (0.31) | 0.59 (0.26) | 0.58 (0.27) | 0.70 (0.27) | 0.66 (0.24) | 0.21 (0.06) | | 0.16 (0.04) | 0.16 (0.04) |
| *median (IQR)* | 0.64 (0.50, 0.76) | 0.56 (0.44, 0.68) | 0.54 (0.44, 0.68) | 0.67 (0.56, 0.83) | 0.63 (0.52, 0.75) | 0.20 (0.17, 0.23) | | 0.16 (0.13, 0.19) | 0.16 (0.14, 0.18) |
| Placebo  *mean (SD)* | 0.68 (0.29) | 0.67 (0.61) | 0.56 (0.22) | 0.69 (0.26) | 0.66 (0.26) | 0.20 (0.05) | | 0.17 (0.05) | 0.18 (0.06) |
| *median (IQR)* | 0.63 (0.51, 0.78) | 0.56 (0.44, 0.70) | 0.53 (0.42, 0.67) | 0.65 (0.55, 0.81) | 0.63 (0.51, 0.75) | 0.19 (0.16, 0.23) | | 0.16 (0.14, 0.20) | 0.18 (0.14, 0.21) |
| **Eicosenoic acid** |  |  |  |  |  |  | |  |  |
| DHA *mean (SD)* | 0.47 (0.30) | 0.44 (0.33) | 0.41 (0.25) | 0.39 (0.29) | 0.40 (0.29) | 0.72 (0.19) | | 0.29 (0.08) | 0.25 (0.07) |
| *median (IQR)* | 0.39 (0.30, 0.54) | 0.34 (0.26, 0.49) | 0.35 (0.27, 0.46) | 0.31 (0.23, 0.45) | 0.31 (0.24, 0.45) | 0.74 (0.65, 0.83) | | 0.28 (0.25, 0.32) | 0.23 (0.21, 0.27) |
| Placebo  *mean (SD)* | 0.49 (0.33) | 0.45 (0.30) | 0.42 (0.22) | 0.37 (0.26) | 0.41 (0.35) | 0.71 (0.16) | | 0.28 (0.06) | 0.26 (0.09) |
| *median (IQR)* | 0.40 (0.28, 0.59) | 0.35 (0.27, 0.54) | 0.36 (0.28, 0.51) | 0.30 (0.21, 0.43) | 0.32 (0.23, 0.47) | 0.69 (0.63, 0.80) | | 0.27 (0.24, 0.31) | 0.23 (0.21, 0.27) |
| Eicosadienoic acid |  |  |  |  |  |  | |  |  |
| DHA *mean (SD)* | 0.33 (0.18) | 0.36 (0.34) | 0.35 (0.19) | 0.38 (0.55) | 0.27 (0.19) | 0.53 (0.69) | | 0.21 (0.23) | 0.16 (0.22) |
| *median (IQR)* | 0.30 (0.20, 0.40) | 0.29 (0.20, 0.42) | 0.31 (0.21, 0.44) | 0.28 (0.18, 0.47) | 0.23 (0.15, 0.34) | 0.08 (0.06, 1.30) | | 0.06 (0.05, 0.41) | 0.05 (0.04, 0.30) |
| Placebo  *mean (SD)* | 0.38 (0.28) | 0.32 (0.20) | 0.35 (0.27) | 0.36 (0.25) | 0.30 (0.25) | 0.69 (0.76) | | 0.26 (0.23) | 0.20 (0.19) |
| *median (IQR)* | 0.31 (0.21, 0.47) | 0.28 (0.19, 0.39) | 0.28 (0.20, 0.42) | 0.29 (0.21, 0.44) | 0.25 (0.16, 0.36) | 0.10 (0.07, 1.30) | | 0.10 (0.06, 0.49) | 0.07 (0.04, 0.39) |
| **Henicosaenoic acid** |  |  |  |  |  |  | |  |  |
| DHA *mean (SD)* | 0.37 (0.30) | 0.34 (0.30) | 0.32 (0.21) | 0.38 (0.38) | 0.27 (0.18) | 0.92 (0.72) | | 0.37 (0.28) | 0.29 (0.19) |
| *median (IQR)* | 0.28 (0.19, 0.45) | 0.27 (0.17, 0.40) | 0.27 (0.18, 0.41) | 0.27 (0.17, 0.46) | 0.23 (0.15, 0.34) | 1.15 (0.04, 1.49) | | 0.41 (0.03, 0.57) | 0.36 (0.04, 0.43) |
| Placebo  *mean (SD)* | 0.39 (0.27) | 0.40 (0.62) | 0.32 (0.20) | 0.40 (0.39) | 0.29 (0.24) | 0.77 (0.73) | | 0.28 (0.25) | 0.21 (0.18) |
| *median (IQR)* | 0.29 (0.20, 0.50) | 0.27 (0.18, 0.40) | 0.26 (0.19, 0.40) | 0.29 (0.18, 0.43) | 0.23 (0.14, 0.34) | 0.90 (0.03, 1.43) | | 0.34 (0.02, 0.49) | 0.24 (0.02, 0.37) |
| Dihomo gamma linoleic acid, n6 |  |  |  |  |  |  | |  |  |
| DHA *mean (SD)* | 0.38 (0.28) | 0.35 (0.22) | 0.42 (0.32) | 0.47 (0.67) | 0.29 (0.30) | 0.83 (0.28) | | 0.66 (0.17) | 0.42 (0.17) |
| *median (IQR)* | 0.31 (0.22, 0.47) | 0.29 (0.19, 0.44) | 0.32 (0.21, 0.48) | 0.31 (0.19, 0.50) | 0.22 (0.15, 0.33) | 0.77 (0.64, 1.00) | | 0.65 (0.56, 0.76) | 0.38 (0.31, 0.49) |
| Placebo  *mean (SD)* | 0.40 (0.26) | 0.36 (0.24) | 0.39 (0.30) | 0.40 (0.42) | 0.29 (0.19) | 0.86 (0.24) | | 0.72 (0.21) | 0.42 (0.11) |
| *median (IQR)* | 0.33 (0.23, 0.48) | 0.31 (0.21, 0.42) | 0.32 (0.20, 0.44) | 0.28 (0.19, 0.42) | 0.22 (0.16, 0.37) | 0.85 (0.69, 0.97) | | 0.71 (0.59, 0.85) | 0.43 (0.35, 0.49) |
| **Arachidonic acid, n6** |  |  |  |  |  |  | |  |  |
| DHA *mean (SD)* | 5.35 (4.05) | 5.68 (3.64) | 7.82 (3.90) | 9.89 (4.10) | 8.81 (4.23) | 0.83 (0.21) | | 0.51 (0.12) | 0.49 (0.14) |
| *median (IQR)* | 3.59 (2.18, 8.53) | 4.93 (2.54, 8.92) | 8.16 (4.09, 11.20) | 10.40 (6.52, 13.50) | 8.94 (4.95, 12.80) | 0.78 (0.68, 0.97) | | 0.49 (0.43, 0.58) | 0.47 (0.40, 0.55) |
| Placebo  *mean (SD)* | 5.50 (4.15) | 6.47 (4.12) | 8.78 (4.12) | 11.00 (4.10) | 9.03 (4.46) | 0.91 (0.20) | | 0.54 (0.14) | 0.53 (0.12) |
| *median (IQR)* | 3.98 (2.21, 8.57) | 6.03 (2.62, 9.96) | 9.83 (4.83, 12.00) | 11.60 (8.41, 14.00) | 8.76 (4.95, 13.50) | 0.88 (0.78, 1.01) | | 0.52 (0.44, 0.63) | 0.52 (0.43, 0.62) |
| **Eicosatrienoic acid, n3** |  |  |  |  |  |  | |  |  |
| DHA *mean (SD)* | 0.75 (0.72) | 0.67 (0.39) | 0.70 (0.50) | 0.72 (0.79) | 0.86 (0.90) | 0.04 (0.07) | | 0.02 (0.01) | 0.03 (0.02) |
| *median (IQR)* | 0.63 (0.46, 0.87) | 0.58 (0.41, 0.82) | 0.59 (0.41, 0.83) | 0.58 (0.41, 0.81) | 0.53 (0.29, 1.04) | 0.03 (0.02, 0.04) | | 0.02 (0.01, 0.02) | 0.02 (0.01, 0.03) |
| Placebo  *mean (SD)* | 0.77 (0.44) | 0.72 (0.59) | 0.73 (0.88) | 0.66 (0.50) | 0.77 (0.78) | 0.03 (0.02) | | 0.02 (0.01) | 0.02 (0.02) |
| *median (IQR)* | 0.66 (0.49, 0.93) | 0.58 (0.43, 0.85) | 0.57 (0.39, 0.85) | 0.57 (0.39, 0.78) | 0.53 (0.28, 0.92) | 0.02 (0.02, 0.04) | | 0.02 (0.01, 0.02) | 0.02 (0.01, 0.03) |
| **Behenic acid,** |  |  |  |  |  |  | |  |  |
| DHA *mean (SD)* | 1.44 (1.13) | 1.34 (0.88) | 1.28 (0.89) | 1.23 (0.77) | 1.23 (1.36) | 0.09 (0.05) | | 0.07 (0.04) | 0.09 (0.07) |
| *median (IQR)* | 1.25 (0.83, 1.67) | 1.18 (0.81, 1.61) | 1.14 (0.76, 1.60) | 1.06 (0.78, 1.53) | 0.82 (0.22, 1.84) | 0.10 (0.04, 0.12) | | 0.07 (0.04, 0.09) | 0.09 (0.06, 0.10) |
| Placebo  *mean (SD)* | 1.53 (1.32) | 1.40 (1.08) | 1.29 (1.23) | 1.23 (0.86) | 1.20 (1.48) | 0.09 (0.05) | | 0.07 (0.04) | 0.09 (0.07) |
| *median (IQR)* | 1.23 (0.87, 1.79) | 1.15 (0.79, 1.68) | 1.08 (0.74, 1.53) | 1.07 (0.79, 1.47) | 0.59 (0.22, 1.80) | 0.09 (0.04, 0.13) | | 0.07 (0.04, 0.09) | 0.08 (0.05, 0.11) |
| **Eicosapentaenoic acid** |  |  |  |  |  |  | |  |  |
| DHA *mean (SD)* | 0.46 (0.42) | 0.38 (0.29) | 0.38 (0.26) | 0.38 (0.32) | 0.32 (0.18) | 0.05 (0.04) | | 0.05 (0.02) | 0.05 (0.03) |
| *median (IQR)* | 0.34 (0.21, 0.54) | 0.29 (0.19, 0.47) | 0.30 (0.20, 0.49) | 0.29 (0.17, 0.45) | 0.28 (0.19, 0.42) | 0.05 (0.03, 0.06) | | 0.04 (0.03, 0.06) | 0.04 (0.03, 0.06) |
| Placebo  *mean (SD)* | 0.48 (0.40) | 0.39 (0.30) | 0.37 (0.27) | 0.37 (0.29) | 0.34 (0.22) | 0.05 (0.03) | | 0.05 (0.03) | 0.05 (0.04) |
| *median (IQR)* | 0.35 (0.20, 0.63) | 0.31 (0.20, 0.46) | 0.31 (0.20, 0.44) | 0.29 (0.18, 0.46) | 0.29 (0.20, 0.41) | 0.05 (0.03, 0.06) | | 0.05 (0.03, 0.06) | 0.04 (0.03, 0.06) |
| **Erucic acid** |  |  |  |  |  |  | |  |  |
| DHA *mean (SD)* | 0.46 (0.38) | 0.40 (0.25) | 0.39 (0.25) | 0.40 (0.30) | 0.30 (0.18) | 0.19 (0.06) | | 0.06 (0.02) | 0.06 (0.03) |
| *median (IQR)* | 0.34 (0.23, 0.55) | 0.35 (0.23, 0.53) | 0.34 (0.24, 0.49) | 0.32 (0.21, 0.47) | 0.27 (0.18, 0.37) | 0.20 (0.17, 0.23) | | 0.06 (0.05, 0.07) | 0.05 (0.04, 0.06) |
| Placebo  *mean (SD)* | 0.46 (0.33) | 0.42 (0.30) | 0.39 (0.22) | 0.38 (0.27) | 0.32 (0.18) | 0.20 (0.05) | | 0.06 (0.01) | 0.08 (0.18) |
| *median (IQR)* | 0.36 (0.25, 0.58) | 0.34 (0.23, 0.54) | 0.33 (0.24, 0.49) | 0.31 (0.21, 0.49) | 0.28 (0.19, 0.40) | 0.19 (0.16, 0.22) | | 0.06 (0.05, 0.07) | 0.05 (0.04, 0.06) |
| **Docosadienoic acid** |  |  |  |  |  |  | |  |  |
| DHA *mean (SD)* | 0.44 (0.23) | 0.41 (0.26) | 0.45 (0.29) | 0.44 (0.28) | 0.33 (0.19) | 0.25 (0.07) | | 0.08 (0.03) | 0.06 (0.03) |
| *median (IQR)* | 0.39 (0.28, 0.54) | 0.36 (0.24, 0.52) | 0.40 (0.26, 0.55) | 0.38 (0.26, 0.54) | 0.29 (0.20, 0.40) | 0.26 (0.22, 0.30) | | 0.08 (0.06, 0.10) | 0.05 (0.04, 0.06) |
| Placebo  *mean (SD)* | 0.44 (0.26) | 0.44 (0.28) | 0.45 (0.29) | 0.44 (0.27) | 0.36 (0.27) | 0.26 (0.09) | | 0.08 (0.02) | 0.06 (0.02) |
| *median (IQR)* | 0.38 (0.28, 0.58) | 0.37 (0.25, 0.53) | 0.37 (0.26, 0.55) | 0.37 (0.24, 0.56) | 0.27 (0.20, 0.44) | 0.25 (0.20, 0.32) | | 0.08 (0.06, 0.09) | 0.05 (0.04, 0.07) |
| **Docosatetraenoic acid** |  |  |  |  |  |  | |  |  |
| DHA *mean (SD)* | 0.59 (0.31) | 0.56 (0.37) | 0.58 (0.49) | 0.51 (0.29) | 0.57 (0.28) | 0.04 (0.03) | | 0.02 (0.01) | 0.03 (0.02) |
| *median (IQR)* | 0.54 (0.37, 0.77) | 0.48 (0.32, 0.69) | 0.54 (0.34, 0.72) | 0.48 (0.32, 0.63) | 0.52 (0.38, 0.69) | 0.03 (0.03, 0.05) | | 0.02 (0.02, 0.03) | 0.02 (0.02, 0.03) |
| Placebo  *mean (SD)* | 0.60 (0.32) | 0.55 (0.47) | 0.54 (0.34) | 0.52 (0.35) | 0.55 (0.24) | 0.05 (0.04) | | 0.02 (0.01) | 0.03 (0.01) |
| *median (IQR)* | 0.58 (0.38, 0.75) | 0.49 (0.34, 0.67) | 0.48 (0.35, 0.67) | 0.47 (0.33, 0.64) | 0.51 (0.38, 0.67) | 0.04 (0.03, 0.05) | | 0.02 (0.02, 0.03) | 0.02 (0.02, 0.03) |
| Tricosaenoic acid |  |  |  |  |  |  | |  |  |
| DHA *mean (SD)* | 1.61 (1.18) | 1.44 (0.96) | 1.72 (0.93) | 2.36 (1.06) | 1.79 (0.96) | 0.52 (0.23) | | 0.14 (0.06) | 0.12 (0.10) |
| *median (IQR)* | 1.14 (0.70, 2.42) | 1.18 (0.69, 2.00) | 1.68 (0.85, 2.48) | 2.41 (1.44, 3.16) | 1.75 (0.87, 2.56) | 0.47 (0.35, 0.67) | | 0.13 (0.11, 0.16) | 0.11 (0.09, 0.12) |
| Placebo  *mean (SD)* | 1.56 (1.13) | 1.94 (1.22) | 2.39 (1.26) | 2.77 (1.16) | 1.98 (1.09) | 0.61 (0.26) | | 0.16 (0.05) | 0.13 (0.04) |
| *median (IQR)* | 1.05 (0.73, 2.07) | 1.61 (0.86, 2.95) | 2.53 (1.17, 3.43) | 2.91 (1.76, 3.66) | 1.77 (0.97, 2.91) | 0.57 (0.46, 0.74) | | 0.15 (0.13, 0.19) | 0.13 (0.10, 0.16) |
| **Docosapentaenoic acid** |  |  |  |  |  |  | |  |  |
| DHA *mean (SD)* | 0.67 (0.47) | 0.97 (1.27) | 0.83 (0.36) | 1.20 (0.62) | 0.70 (0.43) | 0.28 (0.13) | | 0.17 (0.10) | 0.13 (0.10) |
| *median (IQR)* | 0.56 (0.38, 0.83) | 0.75 (0.48, 1.23) | 0.84 (0.53, 1.11) | 1.13 (0.68, 1.55) | 0.66 (0.43, 0.91) | 0.26 (0.19, 0.32) | | 0.15 (0.09, 0.27) | 0.10 (0.06, 0.19) |
| Placebo  *mean (SD)* | 0.79 (0.76) | 0.93 (0.75) | 0.79 (0.43) | 1.62 (0.82) | 0.71 (0.36) | 0.22 (0.09) | | 0.08 (0.04) | 0.06 (0.03) |
| *median (IQR)* | 0.59 (0.38, 0.95) | 0.74 (0.45, 1.22) | 0.74 (0.50, 0.99) | 1.55 (0.96, 2.23) | 0.63 (0.42, 0.96) | 0.20 (0.15, 0.27) | | 0.07 (0.06, 0.10) | 0.06 (0.05, 0.08) |
| **Lignoceric acid** |  |  |  |  |  |  | |  |  |
| DHA *mean (SD)* | 8.18 (2.26) | 7.66 (2.10) | 7.09 (1.88) | 6.99 (1.61) | 6.72 (1.58) | 0.22 (0.08) | | 0.10 (0.03) | 0.10 (0.10) |
| *median (IQR)* | 8.23 (6.48, 10.00) | 7.35 (6.09, 9.11) | 6.69 (5.69, 8.45) | 6.65 (5.89, 7.86) | 6.48 (5.55, 7.59) | 0.21 (0.17, 0.28) | | 0.10 (0.08, 0.12) | 0.09 (0.07, 0.11) |
| Placebo  *mean (SD)* | 8.12 (2.40) | 7.28 (2.08) | 6.88 (1.90) | 6.55 (1.47) | 6.54 (1.40) | 0.24 (0.11) | | 0.10 (0.03) | 0.10 (0.04) |
| *median (IQR)* | 8.09 (6.52, 9.78) | 6.98 (5.94, 8.40) | 6.35 (5.62, 8.03) | 6.31 (5.54, 7.54) | 6.32 (5.42, 7.48) | 0.22 (0.16, 0.29) | | 0.09 (0.08, 0.10) | 0.09 (0.08, 0.12) |
| **Docosapentaenoic acid, n3** |  |  |  |  |  |  | |  |  |
| DHA *mean (SD)* | 0.68 (0.36) | 0.67 (0.45) | 0.72 (0.34) | 0.53 (0.26) | 0.65 (0.33) | 0.15 (0.06) | | 0.09 (0.04) | 0.09 (0.04) |
| *median (IQR)* | 0.62 (0.38, 0.92) | 0.57 (0.41, 0.81) | 0.67 (0.44, 0.96) | 0.48 (0.35, 0.65) | 0.61 (0.38, 0.91) | 0.15 (0.10, 0.17) | | 0.08 (0.06, 0.11) | 0.08 (0.06, 0.10) |
| Placebo  *mean (SD)* | 0.73 (0.43) | 0.70 (0.37) | 0.86 (0.43) | 0.54 (0.27) | 0.75 (0.41) | 0.16 (0.07) | | 0.09 (0.04) | 0.09 (0.04) |
| *median (IQR)* | 0.66 (0.44, 0.93) | 0.65 (0.42, 0.88) | 0.83 (0.51, 1.15) | 0.46 (0.34, 0.69) | 0.68 (0.39, 1.04) | 0.15 (0.10, 0.21) | | 0.09 (0.06, 0.12) | 0.09 (0.06, 0.11) |
| **Nervonic acid** |  |  |  |  |  |  | |  |  |
| DHA *mean (SD)* | 4.59 (1.18) | 4.71 (1.11) | 3.92 (0.95) | 3.99 (0.86) | 4.34 (1.01) | 0.31 (0.11) | | 0.06 (0.04) | 0.05 (0.04) |
| *median (IQR)* | 4.69 (4.09, 5.32) | 4.76 (4.15, 5.35) | 3.80 (3.32, 4.51) | 3.93 (3.47, 4.53) | 4.38 (3.86, 4.90) | 0.32 (0.26, 0.39) | | 0.06 (0.05, 0.06) | 0.04 (0.03, 0.05) |
| Placebo  *mean (SD)* | 4.61 (1.25) | 4.55 (1.10) | 3.86 (0.84) | 3.98 (0.89) | 4.26 (0.88) | 0.33 (0.12) | | 0.06 (0.02) | 0.05 (0.04) |
| *median (IQR)* | 4.73 (3.97, 5.39) | 4.57 (3.97, 5.27) | 3.82 (3.30, 4.42) | 3.92 (3.41, 4.45) | 4.26 (3.87, 4.77) | 0.33 (0.25, 0.42) | | 0.06 (0.05, 0.07) | 0.04 (0.03, 0.06) |
| Baseline DHA (n=292), Placebo (n=256); Delivery, DHA (n=308), Placebo (n=279); 6 month postpartum, DHA (n=296), Placebo (n=268) | | | | | | |  |  |  |
| Cord Blood: DHA (n=304), Placebo (n=271); Venous blood at age 12 month: DHA (n=266), Placebo (n=241) | | | | | | |  |  |  |
| Breast milk: DHA (n=60), Placebo (n=60) at each time-point | | | | | | |  |  |  |

Figure S1: Flowchart for the number of maternal blood, infant blood and breast milk samples analysed at different time points in the DHANI Cohort


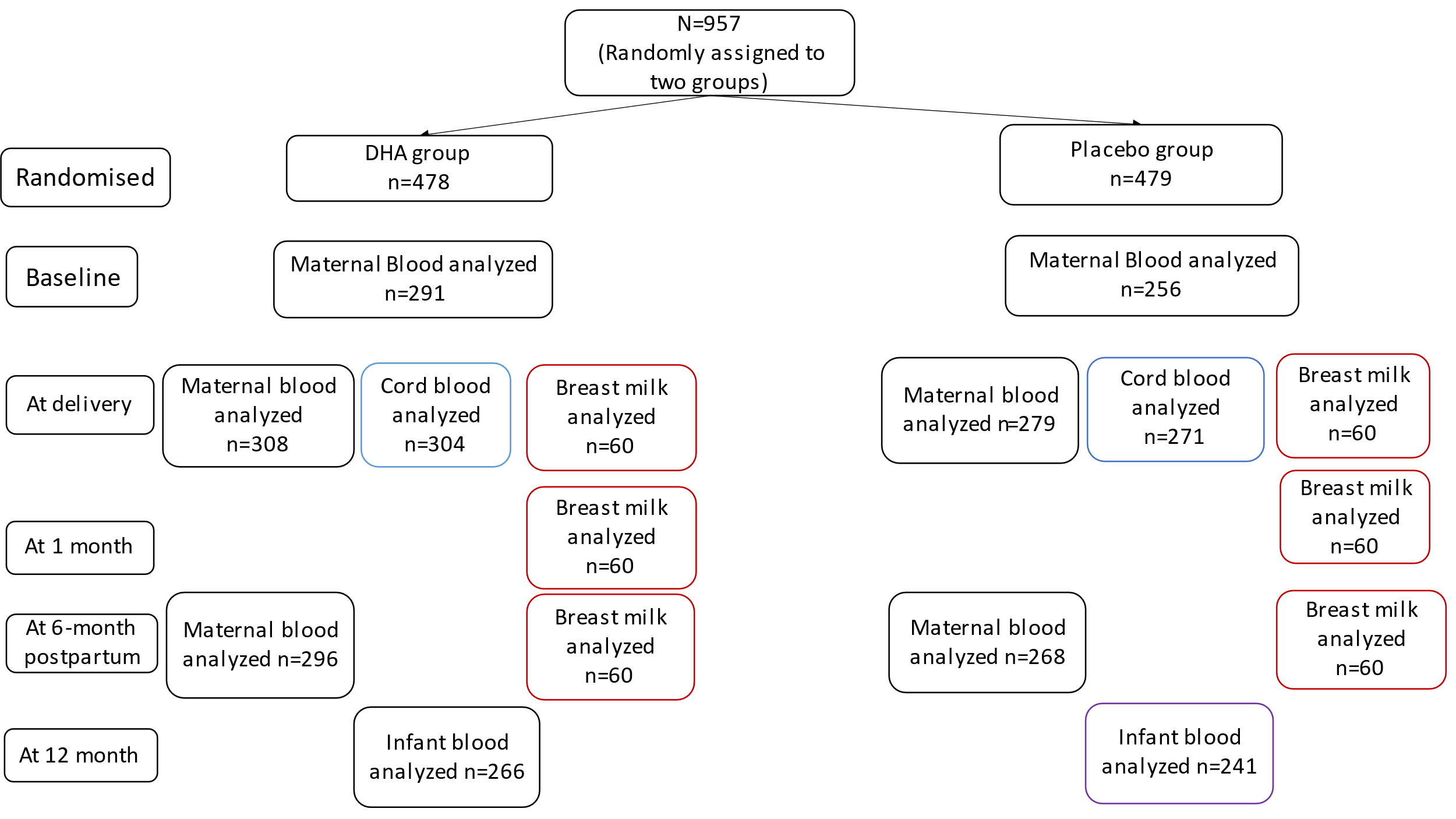


Figure S2: Mean Arachidonic acid values with a 95 % Confidence interval over the time period

|  |
| --- |
|  |
| *Solid shapes for the DHA group, Hollow shapes for the Placebo group* |

Figure S3: Mean Omega-6 values with a 95 % Confidence interval over the time period

|  |
| --- |
|  |
|   *Solid shapes for the DHA group, Hollow shapes for the Placebo group* |

Figure S4: Mean Omega-3 values with a 95 % Confidence interval over the time period

|  |
| --- |
|  |
|  |

*Solid shapes for the DHA group, Hollow shapes for the Placebo group*
